# Assessment of Primary Care Clinician Concordance With Guidelines for Use of Magnetic Resonance Imaging in Patients With Nonspecific Low Back Pain in the Veterans Affairs Health System

**DOI:** 10.1001/jamanetworkopen.2020.10343

**Published:** 2020-07-13

**Authors:** Paul G. Barnett, Josephine C. Jacobs, Jeffrey G. Jarvik, Roger Chou, Derek Boothroyd, Jeanie Lo, Andrea Nevedal

**Affiliations:** 1 Veterans Affairs Health Economics Resource Center, VA Palo Alto Health Care System, Menlo Park, California; 2 Center for Innovation to Implementation, VA Palo Alto Health Care System, Menlo Park, California; 3 Department of Radiology, University of Washington, Seattle; 4 Department of Neurological Surgery, University of Washington, Seattle; 5 Department of Health Services, University of Washington, Seattle; 6 Department of Clinical Epidemiology and Medical Informatics, Oregon Health & Science University, Portland; 7 Department of Medicine, Oregon Health & Science University, Portland; 8 Quantitative Research Unit, Stanford University Medical School, Stanford, California

## Abstract

**Importance:**

Magnetic responance imaging (MRI) of the lumbar spine that is not concordant with treatment guidelines for low back pain represents an unnecessary cost for US health plans and may be associated with adverse effects. Use of MRI in the US Department of Veterans Affairs (VA) primary care clinics remains unknown.

**Objective:**

To assess the use of MRI scans during the first 6 weeks (early MRI scans) of episodes of nonspecific low back pain in VA primary care sites and to determine if historical concordance can identify clinicians and sites that are the least concordant with guidelines.

**Design, Setting, and Participants:**

Retrospective cohort study of electronic health records from 944 VA primary care sites from the 3 years ending in 2016. Data were analyzed between January 2017 and August 2019. Participants were patients with new episodes of nonspecific low back pain and the primary care clinicians responsible for their care.

**Exposures:**

MRI scans.

**Main Outcomes and Measures:**

The proportion of early MRI scans at VA primary care clinics was assessed. Clinician concordance with published guidelines over 2 years was used to select clinicians expected to have low concordance in a third year.

**Results:**

A total of 1 285 405 new episodes of nonspecific low back pain from 920 547 patients (mean [SD] age, 56.7 [15.8] years; 93.6% men) were attributed to 9098 clinicians (mean [SD] age, 52.1 [10.1] years; 55.7% women). An early MRI scan of the lumbar spine was performed in 31 132 of the episodes (2.42%; 95% CI, 2.40%-2.45%). Historical concordance was better than a random draw in selecting the 10% of clinicians who were subsequently the least concordant with published guidelines. For primary care clinicians, the area under the receiver operating characteristic curve was 0.683 (95% CI, 0.658-0.701). For primary care sites, the area was under this curve was 0.8035 (95% CI, 0.754-0.855). The 10% of clinicians with the least historical concordance were responsible for just 19.2% of the early MRI scans performed in the follow-up year.

**Conclusions and Relevance:**

VA primary care clinics had low rates of use of early MRI scans. A history of low concordance with imaging guidelines was associated with subsequent low concordance but with limited potential to select clinicians most in need of interventions to implement guidelines.

## Key Points

**Question:**

What are rates of concordance with guidelines for the use of magnetic resonance imaging in patients with nonspecific low back pain among primary care clinicians in the US Department of Veterans Affairs (VA)?

**Findings:**

In this cohort study of 1 285 405 primary care visits of 920 547 patients, an early magnetic resonance imaging scan of the lumbar spine was performed in 2.42% of primary care episodes for uncomplicated low back pain in VA primary care clinics.

**Meaning:**

Results of this study suggest that the use of magnetic resonance imaging in nonspecific low back pain by VA primary care clinicians is lower than rates that have been reported for US patients with commercial insurance.

## Teaser

This cohort study examines the use of magnetic resonance imaging for low back pain in US Department of Veterans Affairs primary care clinics to assess concordance with published guidelines.

## Introduction

Low back pain has been reported to be the second most common reason for physician office visits^1^ and its associated health care costs have been reported to be increasing.^2,3^ Magnetic resonance imaging (MRI) is used routinely, with 8% to 21% of patients with low back pain in US health plans receiving a scan.^4,5,6^ The Joint Clinical Practice Guideline from the American College of Physicians and the American Pain Society suggests that clinicians should not routinely perform MRI imaging during the first 6 weeks of an episode of nonspecific low back pain without indications that serve as a red flag.^7^ An estimated 26% to 44% of MRI scans of the lumbar spine are not guideline concordant.^8,9,10,11^ Consequences of inappropriate imaging include the direct costs of imaging and further costs of associated interventions, often with similar or worse patient outcomes.^12,13,14,15,16^

The goals of this study were to determine if a program to implement guidelines is needed in the Veterans Affairs (VA) health care system and, if so, assess whether particular clinicians or clinics could reliably be identified as having low concordance. We hypothesized that the use of MRI within 6 weeks of a new episode of low back pain (early MRI scan) is at least as great in US Department of Veterans Affairs (VA) primary care clinics as in other parts of the US health care system. We also hypothesized that historical concordance is better than a random draw in selecting clinicians and clinics for a program to improve concordance with guidelines. A selective approach that focuses on the least concordant clinicians could spare the time of those who are concordant with imaging guidelines. The potential lost time is substantial. The Medicare mandate to use computerized decision support for certain advanced imaging orders^17^ represents an estimated annual cost of $123 million of clinician time.^18^ A selective approach also has implications for patient safety. Clinically important alerts may be missed when clinicians receive too many unneeded notifications.^17,19,20^

## Methods

We evaluated care for low back pain in the VA primary care system in the 3 years ending June 30, 2016, with a retrospective analysis of a national repository of extracts of electronic health records. The Administrative Panels for the Protection of Human Subjects of Stanford University approved the study with exemption from informed consent because of use of publicly available deidentified data. Analysis began in January 2017 and was completed in August 2019. This study followed the Strengthening the Reporting of Observational Studies in Epidemiology (STROBE) reporting guideline for cohort studies.

### Cohort

A cohort was constructed of episodes of nonspecific low back pain without any initial indication for MRI. Exclusion criteria included an initial diagnosis of specific back pain, a condition that serves as a red flag, or continuation of care for chronic back pain. Episodes began with a primary care visit for nonspecific pain of the lower back: lumbar sprain, strain, spondylosis, or disk degeneration. Visits characterized exclusively by a diagnosis of unspecified backache or dorsalgia were excluded because these conditions do not necessarily involve the lower back.

Potential index visits were excluded if there was a specific back pain diagnosis: myelopathy, radiculopathy, sciatica, injury to the coccyx, spinal stenosis, or disk herniation, which are possible indications for MRI. Additional visits were excluded if there was an indication that served as a red flag for MRI: trauma in the prior 45 days; lumbar spine surgery in the past 90 days; cancer, neurologic impairment, infections, or injection drug use in the past year; or autoimmune or inflammatory conditions, neoplastic abnormalities, radiation therapy or congenital malformation in the last 5 years.^21^ Exclusions were based on all care provided or purchased by VA primary care clinics (eFigure in the Supplement).

A visit was considered to be the start of a new episode if no health services for thoracic, lumbar, sacral, or sacrococcygeal back pain had been received in the prior 6 months. This interval defined a new episode in previous studies.^6,22^ Patients with prior care for low back pain could initiate a new episode if at least 6 months had elapsed without care for back pain.

Diagnostic codes were selected from a report that compared published definitions of back pain with the resulting count of VA back pain cases^23^ and from the Medicare performance measure for imaging.^21^ Two of us (J.C.J and R.C.) selected codes to exclude episodes in which MRI might have been indicated at the time of the index visit. *International Classification of Diseases, Ninth Revision (ICD-9)* codes were applied to care provided before October 1, 2015, and *International Statistical Classification of Diseases and Related Health Problems, Tenth Revision (ICD-10)* codes were applied after October 1, 2015.

Episodes were attributed to the primary care clinician at the time of the index visit listed in the VA Primary Care Management Model database. When no primary care clinician was assigned, care was attributed to the clinician seen at that visit. When more than 1 clinician was encountered, care was attributed to the physician clinician. If more than 1 physician saw the patient, the episode was excluded.

An MRI of the lumbar spine was identified in the Radiology Extract of the VA Managerial Cost Accounting system and in the VA purchased care database (*Current Procedural Terminology* code 72148 or 72158). Scans performed in the first 6 weeks of an episode were deemed early and not concordant with guidelines.^8^ This measure of concordance used episodes of nonspecific low back pain as the denominator, distinct from Medicare measure of concordance, which used scans of the lumbar spine as the denominator.^23^

Patient characteristics included age, sex (reported as gender), opioid medications and tramadol provided by VA in the previous year, and an indicator of care for low back pain in the previous 24 months. The first numeric pain score recorded on the date of the index visit was categorized on a scale from 0 to 10 (0 indicating no pain, 1-3, mild pain, 4-6, moderate pain, and >7, severe pain), groupings that best represent the interference of pain with daily activities.^24^

Clinician characteristics included training (physician, nurse practitioner, physician assistant, or resident physician), age, sex, and the number of episodes attributed to the clinician. Continuous variables were categorized to form 3 approximately equal sized subgroups of clinicians. Primary care provided at a single location was referred to as a primary care site and was characterized as either hospital based or community based.

### Statistical Analysis

We hypothesized that the proportion of episodes with early MRI at VA was at least as great as in other parts of the US health care system. The 95% CI of the VA proportion was found using the standard error of the binomial distribution.

The second hypothesis was that historical concordance was better than a random draw in selecting clinicians and clinics for a program of de-implementation. Clinician concordance with guidelines in the first 2 study years (2014-2015) was used to select those expected to be among the 10% least concordant in the third year (2016). A nonparametric method was used to define the 95% CI for the area under the receiver operating characteristic curve.^25^ The lower bound of the 95% CI was compared with a threshold of 0.5, the area under the curve of a noninformative test. To address potential bias from differences in patient case mix, historical concordance was risk adjusted with the results of multivariable logistic regression with patient characteristics as the independent variables. Shrinkage adjustment, often used to evaluate hospital performance,^26,27,28,29,30,31,32^ addressed potential loss of reliability due to small sample size (fewer observations for a clinician) and lack of variance (reduced variation between clinicians).^33^ This estimator shrinks less reliable estimates toward the population mean.^34^ The ROC curves were determined for unadjusted, risk adjusted, and shrinkage and risk-adjusted historical concordance. The areas under these curves were compared using the same nonparametric method.^25^ The practical value of selecting clinicians expected to be the least concordant based on their historical concordance was determined by finding the proportion of all early MRI scans in the follow-up year that were received by their patients.

Univariate associations between early MRI and clinician and patient characteristic were examined by finding the proportion of episodes with that characteristic that had an early MRI scan. The 95% CI was estimated from the standard error of the binomial. Because there were multiple independent comparisons, differences in proportions were reported as significant in the text only if *P* < .001.

Multivariable logistic regression was used to consider the association of each clinician and patient factor with an early MRI scan while controlling for all other factors. The regression included crossed random effects for clinician and site, a specification that accommodated clinicians practicing at more than 1 site. Within-patient random effects could not be modeled because too few patients had more than 1 episode.

The intraclass correlation coefficient was determined from a logistic regression with clinician and site random effects and no independent variables.^35^ Explained variance was partitioned to clinician, site, and patient by analysis of a multivariable random-effects logistic regression with patient level independent variables.^35^ All analyses were performed using SAS version 9.2 (SAS Institute Inc). All statistical tests were conducted with 2-tailed tests.

## Results

A total of 1 285 405 episodes of nonspecific low back pain from 920 547 patients in the 3 years ending June 30, 1996, was studied. The mean (SD) patient age at the start of the episode was 56.7 (15.8) years; 93.6% of the cohort were men. Responsibility for these episodes was attributed to 9098 clinicians (mean [SD] clinician age, 52.1 [10.1] years; 55.7% women). Clinician and patient characteristics are found in Table 1.

**Table 1.** Characteristics of Clinicians and Episodes of Uncomplicated Nonspecific Low Back Pain

Characteristic	No. (%)
**Clinicians (N = 9098)**	
Clinician type	
Physician	6442 (70.8)
Nurse practitioner	1846 (20.3)
Physician assistant	629 (6.9)
Resident physician	114 (1.3)
Clinician sex (N = 7095)	
Male	3146 (44.3)
Female	3949 (55.7)
Clinician age, y (N = 7114)	
Mean (SD)	52.1 (10.3)
<50	2799 (39.3)
50-60	2439 (34.3)
>61	1876 (26.4)
Clinician practice size, LBP episodes	148.4 (143.7)
Mean (SD)	
<57	2939 (32.3)
57-171	3097 (34.0)
≥172	3062 (33.7)
Clinician practice location	
Satellite clinics only	2908 (32.0)
Hospital based clinics only	2727 (30.0)
Both satellite and hospital based clinics	3463 (38.1)
Clinician practice period	
Historical period and follow-up year	6302 (69.3)
Historical period only	1770 (19.5)
Follow-up year only	1026 (11.3)
**Episodes (N = 1 285 405)**	
Patient sex	
Male	1 203 509 (93.6)
Female	81 896 (6.4)
Patient age, y	
Mean (SD)	56.7 (15.8)
<40	224 678 (17.5)
41-50	194 237 (15.1)
51-60	268 692 (20.9)
61-70	383 980 (29.9)
≥71	213 818 (16.6)
Pain score at index visit^a^	
Mean (SD)	3.63 (3.14)
No pain	419 271 (32.6)
Mild pain	203 049 (15.8)
Moderate pain	366 848 (28.5)
Severe pain	296 237 (23.0)
Time since last encounter for low back pain, mo	
6-12	524 433 (40.8)
12-24	320 391 (24.9)
>24	440 581 (34.3)
Opioid and tramadol prescriptions in prior 12 mo	
Long-acting opioid	2948 (0.2)
Short-acting opioid	31 039 (2.4)
Tramadol	17 650 (1.4)
None	1 238 822 (96.4)
Clinician during index encounter	
Assigned primary care clinician	1 046 103 (81.4)
Other than assigned primary care clinician	102 906 (8.0)
No assigned primary care clinician	136 396 (10.6)
Clinician practice size, LBP episodes	
Mean (SD)	288.8 (197.4)
<57	66 597 (5.2)
57-171	319 816 (24.9)
≥172	898 992 (69.9)
Type of clinic	
Community based	736 639 (57.3)
Hospital based	548 766 (42.7)
Year of encounter	
2014	423 124 (32.9)
2015	431 528 (33.6)
2016	430 753 (33.5)

Abbreviation: LBP, low back pain.

^a^ The first numeric pain score recorded on the date of the index visit was categorized on a scale from 0 to 10 (0 indicating no pain, 1-3, mild pain, 4-6, moderate pain, and >7, severe pain).

The 1 285 405 episodes represented 92.7% an original sample of 1 387 983 new episodes of nonspecific back pain in VA primary care clinics (eFigure 1 in the Supplement). The final cohort excluded 67 626 episodes without a pain score from the index visit (4.9%), 31 587 additional episodes that could not attributed to a clinician (2.3%), 1832 additional episodes that could not be assigned to a site (0.13%), and 1533 episodes that were missing a patient identifier.

An MRI of the lumbar spine was performed during the first 6 weeks in 31 132 of the 1 285 405 low back pain episodes (2.42%; 95% CI, 2.40%-2.45%; *P* < .001). The hypothesis that an early MRI was performed in at least 4.2% of episodes (the lower bound of the comparable proportion reported outside VA) was rejected.

Table 2 and the eTable in the Supplement show the proportion of episodes with an early MRI for each clinician and patient characteristic. Early MRI scans were less common if the clinician was at least 60 years of age rather than younger than 50 years (odds ratio [OR], 0.861; 95% CI, 0.805-0.922), was responsible for at least 172 episodes over 3 years rather than less than 57 episodes (OR, 0.769; 95% CI, 0.719-0.824), if the index visit was at a community clinic rather than a hospital-based clinic (OR, 0.848; 95% CI, 0.813-0.884), or if the episode occurred in 2016 rather than 2014 (OR, 0.948; 95% CI, 0.920-0.976).

**Table 2.** Episodes of Low Back Pain With an Early MRI Scan^a^

Variable	Episodes with MRI within 42 d of index visit, % (95% CI)
Clinician type	
Physician	2.34 (2.31-2.37)
Nurse practitioner	2.62 (2.55-2.69)
Physician assistant	2.92 (2.80-3.04)
Resident physician	2.69 (2.36-3.02)
Clinician sex	
Male	2.32 (2.28-2.36)
Female	2.47 (2.42-2.51)
Clinician age, y	
<50	2.66 (2.61-2.72)
50-60	2.33 (2.28-2.38)
>61	2.31 (2.26-2.37)
Clinician practice size, LBP episodes	
<57	2.95 (2.82-3.08)
57-171	2.90 (2.85-2.96)
≥172	2.21 (2.18-2.24)
Type of clinic	
Community based	2.67 (2.63-2.72)
Hospital based	2.23 (2.20-2.27)
Patient sex	
Male	2.40 (2.38-2.43)
Female	2.70 (2.59-2.81)
Patient age, y	
<40	3.41 (3.33-3.48)
41-50	3.05 (2.98-3.13)
51-60	2.62 (2.56-2.68)
61-70	2.07 (2.03-2.12)
≥71	1.19 (1.15-1.24)
Pain category at index visit	
No pain	1.00 (0.97-1.03)
Mild pain	2.17 (2.11-2.23)
Moderate pain	2.90 (2.84-2.95)
Severe pain	4.03 (3.96-4.10)
Time since last encounter for low back pain, mo	
6-12	2.22 (2.18-2.26)
12-24	2.08 (2.03-2.13)
>24	2.91 (2.86-2.96)
Opioid and tramadol prescriptions in prior 12 mo	
Long-acting opioid	1.97 (1.47-2.47)
Short-acting opioid	2.49 (2.31-2.67)
Tramadol	2.49 (2.24-2.75)
None	2.42 (2.39-2.45)
Clinician during index encounter	
Assigned primary care clinician	2.33 (2.30-2.35)
Other than assigned primary care clinician	2.91 (2.81-3.01)
No assigned primary care clinician	2.79 (2.70-2.88)
Year of encounter	
2014	2.43 (2.38-2.48)
2015	2.49 (2.45-2.54)
2016	2.34 (2.30-2.39)
All	2.42 (2.40-2.45)

Abbreviation: MRI, magnetic resonance imaging.

^a^ An early MRI scan is defined as use of MRI scans during the first 6 weeks of episodes of nonspecific low back pain.

Patients were less likely to receive an early scan if they were male rather than female (OR, 0.902; 95% CI, 0.862-0.943) and older than 70 years rather than younger than 40 years (OR, 0.359; 95% CI, 0.342-0.376). Patients were more likely to receive an early scan if they had more severe pain in their index visit rather than no pain (OR, 4.324; 95% CI, 4.167-4.488), if they had no assigned primary care clinician at their index visit compared with those who saw their assigned primary care physician (OR, 1.181; 95% CI, 1.137-1.227), or if they had no visit for back pain in the previous 24 months compared with those who did (OR, 1.233; 95% CI, 1.201-1.265).

The results of the multivariable logistic regression appear in Table 3. Most clinician and patient characteristics identified in the univariate comparisons remained statistically significant. For example, performance of MRI scans was less common if the clinician was responsible for at least 172 episodes over 3 years (OR, 0.903; 95% CI, 0.846-0.964), if the index visit was at a community clinic rather than a hospital-based clinic (OR, 0.857; 95% CI, 0.764-0.962), or if the episode occurred in 2016 rather than 2014 (OR, 0.947; 95% CI, 0.917-0.977). Scans were more common in patients with severe pain (OR, 4.197; 95% CI, 4.044-4.356). In contrast with the univariate result, female patients were less likely to get an early scan (OR, 0.936; 95% CI, 0.895-0.983) and there was no significant association between an early scan and attribution of the episode to a physician assistant, having a primary care clinician, or seeing that clinician at the start of the episode. Clinician age and gender were excluded from the multivariable analysis because these variables were missing for more than 20% of observations.

**Table 3.** Multivariable Logistic Regression of Factors Associated With an Early MRI Scan^a^

Attribute	Odds ratio (95% CI)	*P* value
Clinician type		
Physician	1 [Reference]	
Nurse practitioner	1.020 (0.966 to 1.078)	.45
Physician assistant	1.033 (0.950 to 1.124)	.45
Resident physician	1.226 (0.994 to 1.513)	.06
Clinician practice size, LBP episodes		
<57	1 [Reference]	
57-171	1.042 (0.976 to 1.112)	.23
≥172	0.903 (0.846 to 0.964)	<.01
Type of clinic		
Hospital based	1 [Reference]	
Community based	0.857 (0.764 to 0.962)	<.01
Patient sex		
Male	1 [Reference]	
Female	0.938 (0.895 to 0.983)	<.01
Patient age, y		
<40	1 [Reference]	
41-50	0.895 (0.863 to 0.927)	<.001
51-60	0.745 (0.720 to 0.772)	<.001
61-70	0.638 (0.617 to 0.660)	<.001
≥70	0.399 (0.381 to 0.419)	<.001
Pain category at index visit		
No pain	1 [Reference]	
Mild pain	2.035 (1.947 to 2.126)	<.001
Moderate pain	2.815 (2.712 to 2.923)	<.001
Severe pain	4.197 (4.044 to 4.356)	<.001
Time since last encounter for low back pain, mo		
6-12	1 [Reference]	
12-24	0.952 (0.922 to 0.983)	<.01
>24	1.257 (1.223 to 1.292)	<.001
Opioid and Tramadol prescriptions in prior 12 mo		
None	1 [Reference]	
Long-acting opioid	0.769 (0.588 to 1.007)	.06
Short-acting opioid	0.951 (0.879 to 1.029)	.13
Tramadol	1.101 (0.999 to 1.214)	.07
Clinician during index encounter		
Assigned primary care clinician	1 [Reference]	
Other than assigned primary care clinician	1.048 (1.005 to 1.094)	.14
No assigned primary care clinician	0.998 (0.960 to 1.039)	.92
Year of encounter		
2014	1 [Reference]	
2015	1.030 (1.001 to 1.060)	.03
2016	0.947 (0.917 to 0.977)	<.01

Abbreviation: MRI, magnetic resonance imaging.

^a^ An early MRI scan is defined as use of MRI scans during the first 6 weeks of episodes of nonspecific low back pain.

For clinician and site-level associations with an early MRI scan, the intraclass correlation coefficient was 0.114 for primary care clinicians and 0.089 for primary care sites. Analysis of variance found that 10.4% of variance was explained by patient-level factors, 8.9% by primary care clinician, and 8.7% by site, leaving 71.9% of variance unexplained.

Historical data were used to select clinicians who were expected to be among the 10% least concordant with guidelines in the follow-up period. These least concordant clinicians accounted for 31.5% of the early MRI scans. An early MRI scan was performed in 13.0% of the least concordant episodes, a mean (SD) of 4.6 (4.0) scans per clinician. The ability of historical concordance to correctly select the least concordant clinicians is graphed in a receiver operating characteristic (ROC) curve (Figure, A). The area under this ROC was 0.683 (95% CI, 0.658-0.701; *P* < .001), which is significantly greater than 0.5, the area that would result from a random draw. The area under the ROC curve with risk and shrinkage adjustment (C = 0.697; *P* < .001) was significantly greater than the area under the unadjusted curve. The area under the ROC curve with risk adjustment alone (C = 0.678; *P* = .18) was significantly less than the area under unadjusted curve.

**Figure.**
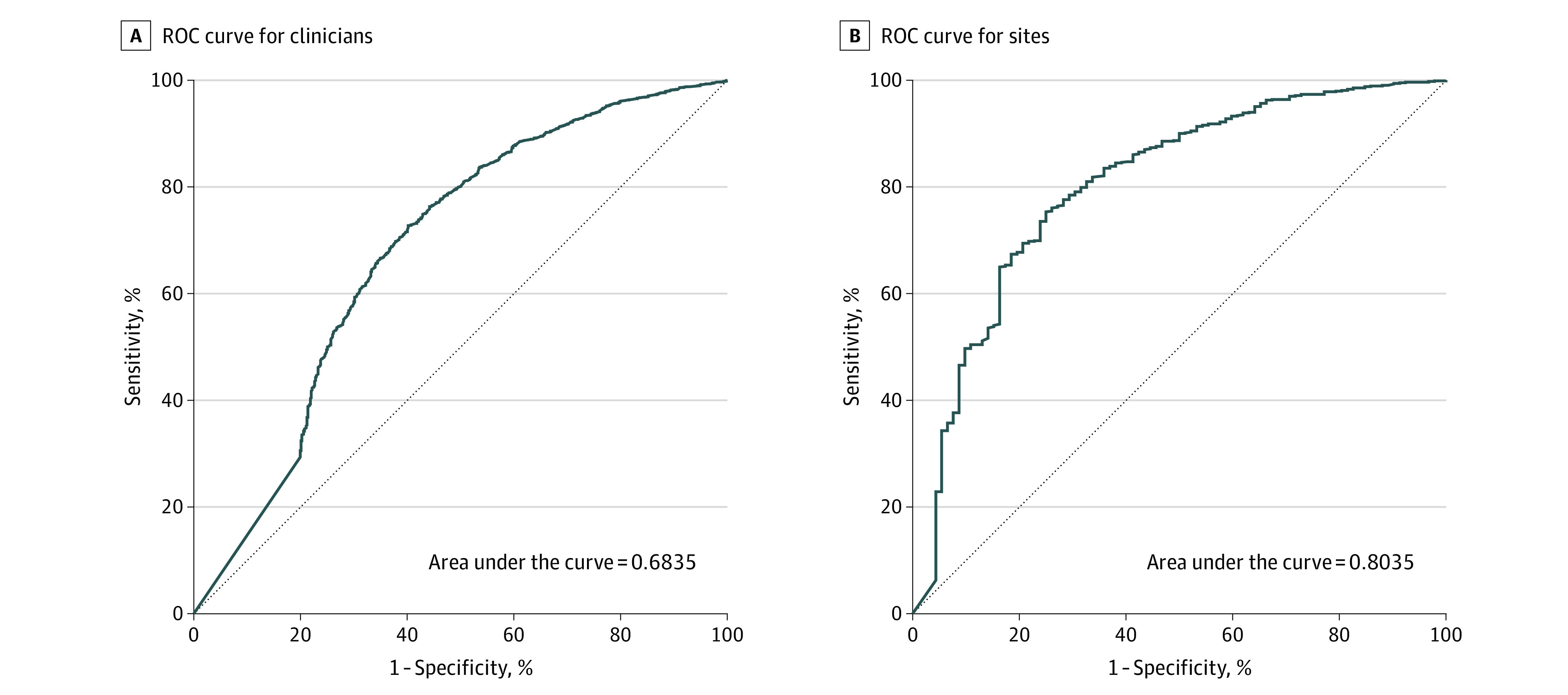
Receiver Operating Characteristic Curves Identifying Low Concordance Based on Historical Concordance Receiver operating characteristic curves based on proportion of episodes of early magnetic resonance imaging scans over 2 years to identify primary care clinicians (A) and primary care sites (B) subsequently determined to be among the 10% least concordant with guidelines for the use of magentic resonance imaging in management of nonspecific low back pain.

Historical data were was also used to select the 10% least concordant primary care sites (Figure, B). An early MRI scan was performed in 7.2% of the episodes of these least concordant sites. They accounted for 15.7% of the scans in the follow-up period. Using historical concordance to select the least concordant sites resulted in the area under ROC of 0.8035 (95% CI, 0.754-0.855; *P* < .001), significantly better than a random draw. There were no significant differences between areas under ROC curves defined without adjustment, with risk adjustment (C = 0.804; *P* = .97), or with shrinkage and risk adjustment (C = 0.810; *P* = .48).

Table 4 presents the results of different decision thresholds along the ROC curve to select the least concordant clinicians. Using historical data to select clinicians expected to be the 10% least concordant accounted for 19.2% of the episodes with early MRI scans in the follow-up period. Using the historical data to select the 10% less concordant sites accounts for 10.3% (1017 of 9883) of the early MRI scans in the follow-up period.

**Table 4.** Early MRI Scans for Low Back Pain Identified by Historical Concordance^a^

Cohort	Follow-up period	
	Early MRI, No. (%)	Episodes of low back pain, No. (%)
**Primary care clinicians with lowest historical concordance**		
5%	995 (10.9)	13 983 (3.5)
10%	1758 (19.2)	30 485 (7.7)
20%	2984 (32.6)	63 998 (16.2)
33%	4570 (49.9)	117 086 (29.6)
50%	6273 (68.5)	193 565 (48.9)
**Primary care sites with lowest historical concordance**		
5%	507 (5.1)	8646 (2.0)
10%	1017 (10.3)	21 938 (5.2)
20%	2581 (26.1)	62 408 (14.8)
33%	3926 (39.7)	105 518 (25.0)
50%	6431 (65.1)	208 682 (49.4)

Abbreviation: MRI, magnetic resonance imaging.

^a^ An early MRI scan is defined as use of MRI scans during the first 6 weeks of episodes of nonspecific low back pain.

Data on clinicians and clinics with episodes only in the follow-up period are not given in the Figure and Table 4. Primary care clinicians without historical data accounted for 14.0% of the clinicians and 9.7% of the early MRI scans in the follow-up year. Primary care sites without historical data accounted for 2.2% of the sites and 2.1% of the early MRI scans in the follow-up year.

## Discussion

Between 8% and 21% of US patients with low back pain receive an MRI of the lumbar spine.^4,5^ Findings from these studies are not comparable with our study as they included patients for whom MRI may be appropriate: those with chronic pain, a specific case of back pain, or an indication that served as a red flag. The VA cohort was limited to new episodes of nonspecific low back pain without an indication that served as a red flag. We determined that 2.42% of these patients received an MRI within the first 6 weeks of their episode, the recommended period for a trial of conservative therapy without using MRI.^7^ The finding was significantly less than the 4.2% found in a comparable study of US patients with commercial insurance.^6^ That study also included a cohort of newly diagnosed patients with nonspecific low back pain without indications that serve as a red flag. An MRI of the lumbar spine was performed in 8.5% of those patients. Although the proportion of episodes with a scan within 6 weeks was not reported, the median time to a scan was 19 days.^6^ The true proportion of patients who received a scan within 6 weeks appears to be between 4.2% (the proportion scanned after 19 days) and 8.5% (the total proportion scanned).

The VA primary care clinicians appear to be more concordant with published practice guidelines for imaging low back pain than other US clinicians. Factors in this concordance may include that VA clinicians are salaried; other US clinicians may have an economic incentive to order scans.^36^ Several VA sites routinely review orders for advanced imaging. In addition, VA may have a lower capacity to conduct MRI.

Historical concordance over the previous 2 years was better than random selection in identifying clinicians and sites that were subsequently found to be among the 10% least concordant with imaging guidelines, but the practical utility of this measure was low. Selecting 10% of primary care clinicians based on historical concordance accounted for 19.2% of early MRI scans in the follow-up period. The low number of scans in VA limits the reliability of the historical concordance as a selection criterion, even with risk and shrinkage adjustment.

### Limitations

This study has limitations. Diagnoses were limited to care provided by or paid for by VA. Diagnostic codes assigned in VA are not subject to review by health care payers and may be less accurate than in other systems. Pain scores in electronic medical records underestimate actual patient pain.^37^ Reliance on this source may have attenuated patient case mix adjustment. We excluded 7.3% of episodes with missing data. Shrinkage adjustment assumes independence of volume and outcomes, but higher volume clinicians were more concordant with guidelines.

## Conclusions

In this study, we found use of MRI to manage nonspecific low back pain by VA primary care clinicians to be more concordant with guidelines than the practice described in a study of patients^6^ with commercial insurance. A history of inappropriate imaging by VA clinicians and clinics was associated with subsequent low concordance, but had limited potential to improve the efficiency of efforts to improve clinician concordance with guidelines. Interventions to improve concordance may be unneeded, given the low utilization of early MRI scans for low back pain in VA.

## Notes

Supplement. **eFigure.** Flow of Visits and Episodes Included in Study and Numbers Excluded **eTable.** Percent of Episodes With an Early MRI, by Attribute of Provider and Patient at Time of Index Visit for Low-back Pain, With Statistical Significance of Comparisons
