# Supplementary material for: Assessment of Primary Care Clinician Concordance With Guidelines for Use of Magnetic Resonance Imaging in Patients With Nonspecific Low Back Pain in the Veterans Affairs Health System
Source: JAMA Netw Open. 2020 Jul 13;3(7):e2010343. doi: 10.1001/jamanetworkopen.2020.10343 (PMC7358914; doi:10.1001/jamanetworkopen.2020.10343)
Supplement: Supplement. — eFigure. Flow of Visits and Episodes Included in Study and Numbers Excluded eTable. Percent of Episodes With an Early MRI, by Attribute of Provider and Patient at Time of Index Visit for Low-back Pain, With Statistical Significance of Comparisons [file jamanetwopen-3-e2010343-s001.pdf]

Supplementary Online Content

Barnett PG, Jacobs JC, Jarvik JG, et al. Assessment of primary care clinician concordance with guidelines for use of magnetic resonance imaging in patients with nonspecific low back pain. JAMA Netw Open. 2020;3(7):e2010343.

doi:10.1001/jamanetworkopen.2020.10343

eFigure. Flow of Visits and Episodes Included in Study and Numbers Excluded

eTable. Percent of Episodes With an Early MRI, by Attribute of Provider and Patient at Time of Index Visit for Low-back Pain, With Statistical Significance of Comparisons

This supplementary material has been provided by the authors to give readers additional information about their work.

© 2020 Barnett PG et al. *JAMA Network Open.*

eFigure. Flow of visits and episodes included in study and numbers excluded

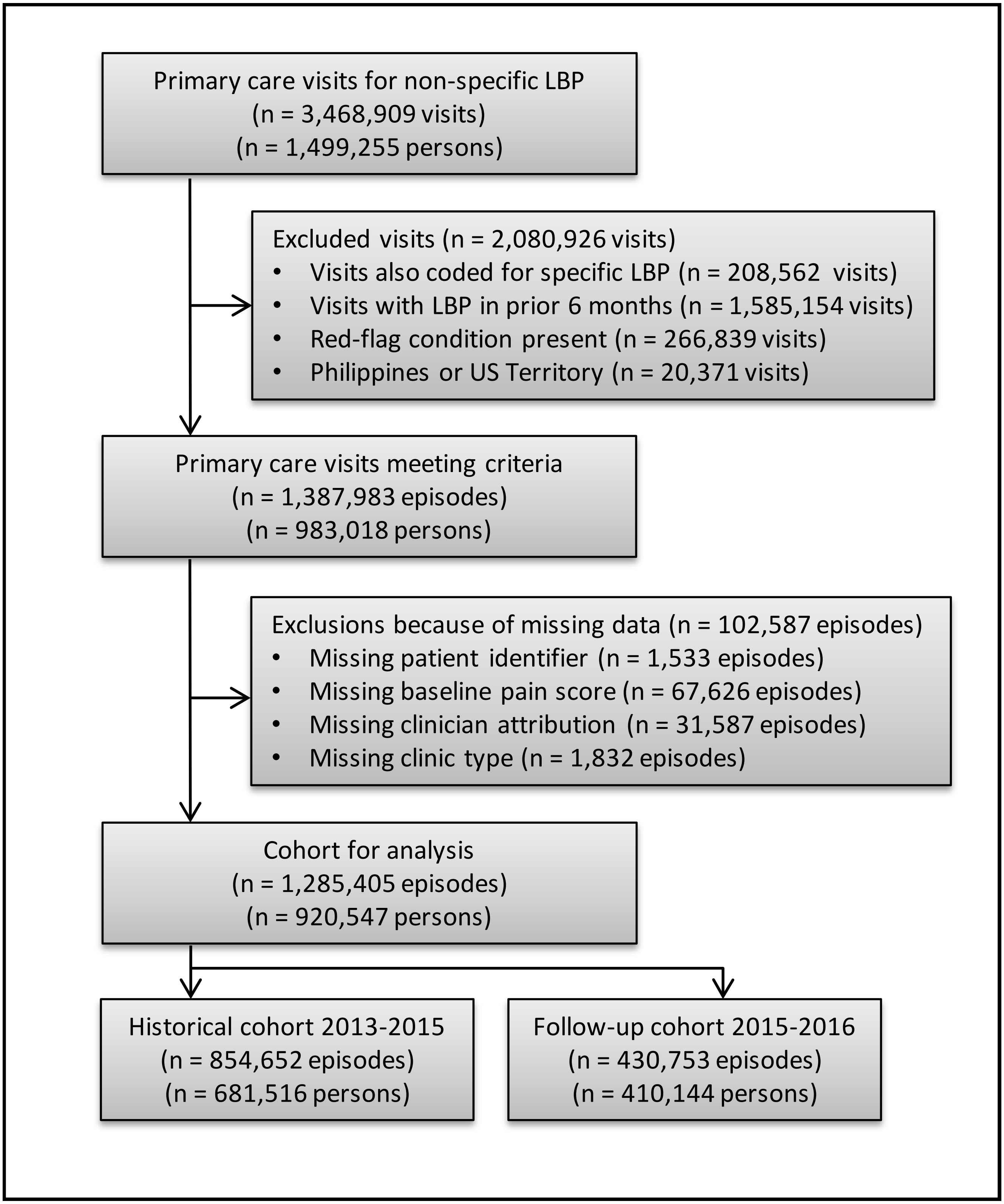

© 2020 Barnett PG et al. *JAMA Network Open.*

eTable. Percent of episodes with an early MRI, by attribute of provider and patient at time of index visit for low-back pain, with statistical significance of comparisons

Attribute	Percent with MRI of lumbar spine within 42 days of index visit	Test of whether values of this variable have significantly different percentage	
Provider Type		MD vs NP	p = .023
Physician	2.34	MD vs PA	p < 0.0001
Nurse Practitioner	2.62	MD vs resident	p = 0.026
Physician's Assistant	2.92	NP vs PA	p =0.011
Resident Physician	2.69	All others comparisons	NS
Provider Gender			
Male	2.32	Male vs. Female	<0.007
Female	2.47		
Provider age			
< 50 years	2.66	<50 vs. 50-60	p < 0.0001
50-60 years	2.33	<50 vs. 60+	p < 0.0001
60+ years	2.31	50-60 vs. 60+	NS
Patient Gender			
Male	2.40	Male vs. female	p < 0.0001
Female	2.70		
Patient Age			
< 40 years	3.41	All pairwise comparisons	p < 0.0001
41-50 years	3.05		
51-60 years	2.62		
61-70 years	2.07		
70+ years	1.19		
Baseline Pain Score			
None	1.00	All pairwise comparisons	p < 0.0001
Mild Pain	2.17		
Moderate Pain	2.90		
Severe Pain	4.03		
Time Since Last Encounter for Low Back Pain			
6 - 12 months	2.22	All pairwise comparisons	p < 0.0001
12 -24 months	2.08		
> 24 months	2.91		
Opioid & Tramadol Prescriptions in Prior 12 Months			
Long-acting opiate	1.97	All pairwise	NS

© 2020 Barnett PG et al. *JAMA Network Open.*

		comparisons	
Short-acting opiate	2.49		
Tramadol	2.49		
None	2.42		
Provider During Index Encounter			
Assigned Primary Care Provider	2.33	PCP vs. did not see PCP	p < 0.0001
Other Than Assigned Primary Care Provider	2.91	PCP vs. no-PCP	p < 0.0001
No Assigned Primary Care Provider	2.79	Did not see PCP vs. no-PCP	NS
Provider practice size, episodes			
< 57 LBP episodes	2.95	< 57 vs. 57-171 patients	p < 0.0001
57-171 LBP episodes	2.90	< 57 vs. 172+ patients	p < 0.0001
172+ LBP episodes	2.21	57-171 vs. 172+ patients	NS
Type of Clinic			
Community Based	2.67	Hospital vs. Satellite	p <.0001
Hospital Based	2.23		
Year of encounter			
2014	2.43	2014-2015	NS
2015	2.49	2014-2016	p = 0.0003
2016	2.34	2015 vs. 2016	p < 0.0001
All	2.42		

© 2020 Barnett PG et al. *JAMA Network Open.*
